# The two faces of pyocyanin - why and how to steer its production?

**DOI:** 10.1007/s11274-023-03548-w

**Published:** 2023-02-18

**Authors:** Joanna Jabłońska, Adrian Augustyniak, Kamila Dubrowska, Rafał Rakoczy

**Affiliations:** 1 Faculty of Chemical Technology and Engineering, Department of Chemical and Process Engineering, West Pomeranian University of Technology in Szczecin, al. Piastów 42, 71-065 Szczecin, Poland; 2 Chair of Building Materials and Construction Chemistry, Technische Universität Berlin, Gustav-Meyer-Allee 25, 13355 Berlin, Germany; 3 Institute of Biology, University of Szczecin, ul. Wąska 13, 71-415 Szczecin, Poland

**Keywords:** Bacterial pigment, Bioprocessing, Phenazines, *Pseudomonas aeruginosa*, Secondary metabolites

## Abstract

The ambiguous nature of pyocyanin was noted quite early after its discovery. This substance is a recognized *Pseudomonas aeruginosa* virulence factor that causes problems in cystic fibrosis, wound healing, and microbiologically induced corrosion. However, it can also be a potent chemical with potential use in a wide variety of technologies and applications, e.g. green energy production in microbial fuel cells, biocontrol in agriculture, therapy in medicine, or environmental protection. In this mini-review, we shortly describe the properties of pyocyanin, its role in the physiology of *Pseudomonas* and show the ever-growing interest in it. We also summarize the possible ways of modulating pyocyanin production. We underline different approaches of the researchers that aim either at lowering or increasing pyocyanin production by using different culturing methods, chemical additives, physical factors (e.g. electromagnetic field), or genetic engineering techniques. The review aims to present the ambiguous character of pyocyanin, underline its potential, and signalize the possible further research directions.

## Key points

- Pyocyanin is not only a virulence factor but also a potent chemical for the industry.
- Pyocyanin production may be stimulated or inhibited depending on the approach.
- A growing interest in pyocyanin motivates the search for novel production methods.

## Pyocyanin – biological origin and properties

*P. aeruginosa* is an opportunistic pathogen often associated with nosocomial infections, cystic fibrosis complications, and arising in antibiotic resistance. However, the adaptability to different environments and production of robust secondary metabolites allowed noticing its biotechnological potential. To date, substances such as rhamnolipids, biopolymers, and pigments have been acquired from *P. aeruginosa* cultures (Bedoya et al. 2019; Mahato et al. 2021). Among pigments, pyocyanin (5-methylphenazin-1-one) is the most studied due to its unique properties. This pigment belongs to the group of phenazines that are heterocyclic compounds containing nitrogen atoms. Pyocyanin (PYO) is a zwitterion at pH 7 and has a low molecular weight, which enables biological membranes’ permeation. It is characterized by a blue-greenish colour at the neutral and alkaline pH that changes to pink-red in acidic conditions. Due to the presence of a phenol group, its characteristic is weakly acidic (pKa 4.9). The pigment’s colour also depends on its redox state. It was reported that oxidized PYO is blue, whereas the reduced form is transparent (Rada and Leto 2013). Thanks to the zwitterionic nature, PYO accepts the electrons from reducing agents, i.e. NADH or reduced glutathione, and transports them to electron acceptors, i.e. oxygen (Liu and Nizet 2009). In oxygen-poor conditions, PYO enables bacterial survival by transporting the electrons away from the cells (Rada and Leto 2013). Therefore, it is utilised by *P. aeruginosa* as a mobile electron transfer and allows it to control the redox balance. PYO leads to the generation of reactive oxygen species (ROS), mostly superoxides (O_2_^∙−^) and hydrogen peroxide (H_2_O_2_). The mechanism of ROS formation by PYO was proposed by Jacob et al. (2011). It states that PYO can be non-enzymatically reduced by reducing agents such as NADH and NADPH which leads to the formation of hydrophenazine. This compound reacts with a second PYO molecule to form two PYO radicals that reduce oxygen molecule to superoxide anion radical (O_2_^·−^). O_2_^·−^ radical can be transformed to H_2_O_2_ by dismutation. The imbalance of ROS leads to oxidative stress that can be a cause of cell death. Koley et al. (2011) reported that *P. aeruginosa* in the biofilm form produces a gradient of PYO in a reduced state called ‘electrocline’. This gradient was proven to extend up to 400 microns and is promoted by the limited presence of an electron acceptor and correlated with the increase in soluble iron. This confirmed that PYO and other phenazines can reduce Fe^3+^ ions to Fe^2+^ under aerobic conditions. PYO and phenazine-1-carboxamide (PCN) are kept within biofilms due to binding to extracellular DNA (eDNA). PYO also promotes eDNA release from the biofilms through cell lysis mediated by H_2_O_2_ (Saunders et al. 2020). The combination of the phenazines and eDNA was also reported to support an efficient extracellular electron transfer.

Phenazines, including PYO, are significant molecules in the physiology, ecological fitness, and competitiveness of *P. aeruginosa* (Mavrodi et al. 2013). PYO’s role is based on its redox properties and plays an important role in polymicrobial communities. Castañeda-Tamez et al. (2018) proved that it restricts the growth of metabolically-redundant bacteria called ‘social cheaters’ that do not contribute to the production of public goods such as siderophores or enzymes. Dietrich et al. (2008) reported that PYO is one of the redox-active molecules that influence colony morphology. Pigment-null mutants produced wrinkled colonies faster than the wild type. Moreover, PYO overproducers remained in the form of smooth colonies throughout the whole experiment. PYO was also confirmed to affect more than 35 genes, excluding the ones in the SoxR regulon (Dietrich et al. 2006). Such a finding underlines the role of PYO in the cell’s physiology. Moreover, Meirelles and Newman (2018) reported that PYO plays a multifaceted role in *P. aeruginosa*. On one hand, it promotes cell survival in biofilms in oxidant-limited conditions. On the other hand, PYO can also lead to autointoxication of the population, resulting in cell death and the release of eDNA. *P. aeruginosa* possesses multiple mechanisms protecting it from PYO, including oxidative stress response mechanisms. Nevertheless, in high concentrations PYO is toxic to *P. aeruginosa* and only some cells called ‘persister-like’ can survive (van den Bergh et al. 2017; Meirelles and Newman 2018).

The virulence of *P. aeruginosa* is controlled by quorum sensing (QS). QS allows the regulation of cell density through the secretion of small autoinducer molecules that while present in certain concentrations activate or repress gene expression. In *P. aeruginosa* two QS systems are based on acyl homoserine lactone signalling (HSL): (1) *las* system and (2) *rhl* system (comprised of the transcriptional activator *RhlR* and *RhlI*) (Pesci et al. 1997; Vilaplana and Marco 2020). *las* system activates *rhlR* and *rhlI*, and therefore it is placed above *rhl* system. Besides HSL signalling, there is a quinolone signalling system (pqs) characteristic of *P. aeruginosa.* It is intertwined with HSL systems. *las* system positively controls the level of quinolone molecule (PQS), while *rhl* system negatively influences PQS levels (Gallagher et al. 2002; Schuster et al. 2003; Brouwer et al. 2014). The biosynthesis of PYO and other phenazines is based on the conversion of chorismate derived from shikimate pathway (da Silva et al. 2021). To date, seven enzymes have been recognized as conserved in all phenazine-producing bacteria – *PhzA*-*PhzG*. It is worth underlining that *P. aeruginosa* has two independent homologous gene clusters, *phzA1B1C1D1E1F1G1* (*phz1*) and *phzA2B2C2D2E2F2G2* (*phz2*) (Mavrodi et al. 2001) that are responsible for phenazine production. These enzymes take part in the conversion of chorismate to phenazine-1-carboxylic acid (PCA) and phenazine-1,6-dicarboxylic acid (PDC). PCA and PDC are recognized as ‘core’ phenazines. PCA is later modified in a strain-specific way to other phenazines. Two genes, *phzM* and *phzS* code two phenazine-modifying enzymes that act together to convert PCA to PYO. Phenazine-1-carboxylate N-methyltransferase produced by *phzM* is essential to provide 5-methylphenazine-1-carboxylate for the final synthesis step employing 1-monooxygenase (expressed by *phzS* gene) that converts it to PYO. Reduced expression of *phzM* creates an oversupply of PCA that may be directly converted by *phzS* to 1-hydroxyphenazine. However, *phzS* alone can also convert PCA to 1-hydroxyphenazine (Mavrodi et al. 2001). It was proven by Dietrich et al. (2006) that PYO is the terminal signalling factor in the QS of *P. aeruginosa.* PYO biosynthesis is linked to QS due to the fact that regulatory proteins comprised in *rhl* and *pqs* systems, namely *RhlR* and *PqsE*, activate both *phz* operons by acting together (Higgins et al. 2018). To date, full biosynthesis pathways of phenazines and pyocyanin have been described and presented, e.g., in works of Mavrodi et al. (2001), Blankenfeldt and Parsons (2014) and da Silva et al. (2021), or in KEGG database (https://www.genome.jp/pathway/map00405, accessed on 13.02.2023).

The clinical significance of PYO, which is often a hallmark of bacteraemia, resulted in the development of many detection methods. Among them, are spectrophotometric reads, voltammetric detection, and high-performance liquid chromatography, often coupled with a mass spectrometer (HPLC-MS). Absorbance reads can be performed either on the culture supernatant or the PYO extracted with chloroform and hydrochloric acid (λ = 520 nm for acidic extract, λ = 690 for extract in chloroform (Vilaplana and Marco 2020)). Thanks to its redox properties, the pigment can also be detected in buffer/medium employing voltammetric methods (Schneider et al. 2022). The most reliable method is HPLC-MS, which not only enables the quantification of PYO, but also verifies the presence of the desired metabolite (Vilaplana and Marco 2020).

## Why is pyocyanin production worth controlling?

**Fig. 1:**
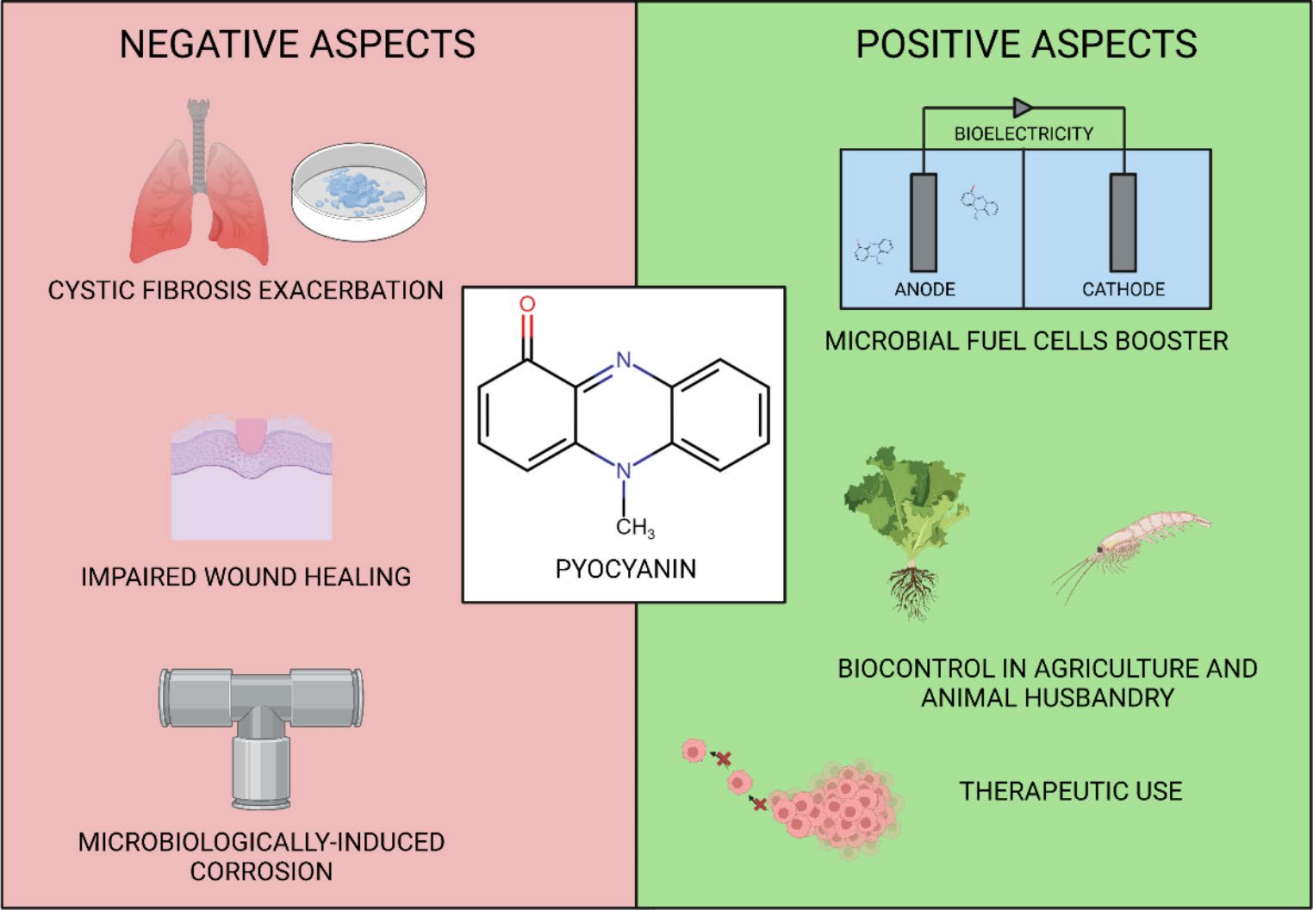
Positive and negative aspects of PYO.

PYO has been called a ‘double-edged sword’ of *P. aeruginosa* for it can have both beneficial and detrimental effects on the producer population (Meirelles and Newman 2018). This problem scales up to humans, animals, and technological systems (see Fig. 1). The negative role of PYO has been extensively studied and described for decades (Liu and Nizet 2009; Rada and Leto 2013; Hall et al. 2016). The most prominent reason behind that is its role in cystic fibrosis (CF). PYO toxicity is based on the generation of ROS. Naturally, ROS occur in vital and normally functioning cells. However, their excess leads to oxidative stress that disturbs the cell’s metabolism, which can eventually cause the cell’s death. PYO oxidizes NADH and NADPH, which, together with increased ROS, enhances the redox potential of cytosol. Another consequence is reduced ATP production and the ratio of reduced to oxidized glutathione. Detrimental effects of PYO were also reported concerning urological, nervous, hepatic, and vascular systems (Hall et al. 2016). Among these are the influence on antioxidant enzymes, the production of interleukin IL-2, IL-6, prostaglandin E2, and immunoglobulin. Moreover, PYO can alter lymphocyte proliferation, macrophage function, ciliary beating, and increase mucous secretion (Bianchi et al. 2008; Hao et al. 2012; McDermott et al. 2013). Ulmer et al. (1990) underlined that PYO effects are dose-dependent. For example, it stimulated the proliferation of T and B lymphocytes, IL-2 production, and B lymphocyte differentiation when applied in low dosages. However, higher concentrations resulted in opposite observations. Peng et al. (2022) analysed the influence of PYO on mice and pig digestive tracts. The authors proved that PYO exposure led to dysbiosis of microbiota and damage to the mucus layer. It was recently reported that PYO can permeate the blood-brain barrier and influence cognitive function in the murine model (Rashid et al. 2022). PYO is also present during *P. aeruginosa* infections of the wounds. It was reported to inhibit the repair of the wound (Muller et al. 2009) by inducing cellular senescence. PYO accelerates neutrophil apoptosis in vivo in mice. This results in reduced local inflammation and supports *P. aeruginosa* survival during the infection (Allen et al. 2005). In contrast to other bacterial pigments, e.g. staphyloxanthin or melanin that have an antioxidant nature, PYO exhibits pro-oxidant properties (Liu and Nizet 2009). From the engineering standpoint, the pivotal role of PYO in microbiologically induced corrosion (MIC) was recently recognized (Huang et al. 2020). TEM analyses showed that *P. aeruginosa* biofilm and PYO led to the breakdown of the passive film of iron oxides and accelerated the MIC process (Li et al. 2022b). Moreover, it was proven by Huang et al. (2020) that mutants with *phzS* and *phzM* gene knockouts unable to produce PYO exhibited a lower potential to support MIC.

On the other side, PYO can be a potent and useful chemical in industry and diagnostics. Microbial fuel cells (MFC) for green energy generation seem to be the most popular application of PYO. Thanks to the unique redox properties it can serve as an electron shuttle which results in higher production of the current that is confirmed by numerous recent works (Bagchi and Behera 2021; Ajunwa et al. 2021, 2022). The redox properties of PYO were also applied in a sensor recording glucose levels (Ohfuji et al. 2004).

PYO can also be potentially useful in agriculture. It exhibits antibacterial and antifungal properties, and it successfully reduced the number of *Xanthomonas oryzae* pv. *oryzae* causing bacterial leaf blight in rice (Yasmin et al. 2017) and *Macrophomina phaseolina* which is the agent of charcoal rot in chickpea (Khare et al. 2011). Furthermore, Gupta et al. (2020) showed that *Pseudomonas* spp. isolated from soil can play a role in peanut growth by inducing plant immune response. There is a commercially available product called Shenqinmycin, which is a phenazine-based antifungal agent (in this case PCA) (Zhao et al. 2018). The potential of PYO is not restricted to plants. This pigment can be applied as a drug for the control of vibriosis in shrimp aquacultures (Balakrishnan et al. 2022). It was proven that LC_50_ values of PYO (concentration needed to reduce cell viability by 50%) for *Penaeus monodon* were higher than the ones required to obtain a bactericidal effect on *Vibrio harveyi* (Priyaja et al. 2017). This underlines the potential use of PYO as a biocontrol agent in animal husbandry.

Moreover, PYO can also be used in environmental protection. The use of PYO in the degradation of various compounds was previously reported. Among them are 2,4,6-trinitrotoluene (Stenuit et al. 2012), phenanthrene (Nie et al. 2020), hexadecane (Nie et al. 2018) and tetrabromobisphenol (Huang et al. 2020b). The mechanism suggested by Stenuit et al. (2012) indicates that PYO coupled with NAD(P)H in aerobic conditions can denitrate 2,4,6-trinitrotoluene (TNT). Since superoxide radical anions were detected, it has been suggested that the underlining mechanism of this phenomenon is based on superoxide-driven nucleophilic attack of the radical on TNT. Similarly, Nie et al. (2020) showed that PYO/NADH/O_2_ system generated reactive oxygen species that led to the cleavage of phenanthrene ring and the formation of phthalate products. These observations describe PYO’s redox nature. Das and Ma (2013) demonstrated that PYO enhanced hydrocarbon emulsification with biosurfactants. However, the mechanism of this phenomenon remains unclear. Wu et al. (2014) presented the dual use of PYO in toluene biodegradation and power generation in MFC. DeBritto et al. (2020) showed that PYO is a durable fabric dye when used in its acidic form. The tested fabric was a cotton cloth that turned pink due to PYO presence and its colour persisted after 3–5 washings with soap.

Saleem et al. (2021) suggested that PYO could be utilised as a food preservative and food-grade colourant after ruling out the potential toxicity to humans. Hamad et al. (2020) confirmed the antifungal and antibacterial properties of PYO, verified the toxicity on brine shrimp and mice, and concluded that no toxic effect was noted for 50 µg/mL and 750 µg/mL, respectively. Li et al. (2018) also reported that PYO is well-tolerated by probiotic microorganisms (*Lactobacillus* spp.). However, considering an opposing conclusion made by Peng et al. (2022) who indicated that PYO may be detrimental to the function of intestinal microbiota, it appears that further experimental investigations still have to be carried out. Regarding humans, the current data cannot exclude that ingesting PYO will cause adverse effects. Since the observations made by different authors are often contradictory, a clinical study may be necessary to reliably define its toxicity.

Even though, PYO is a well-known virulence factor, thanks to its properties it perhaps can find use in medicine. As previously mentioned, PYO exhibits strong antimicrobial properties against fungal, e.g. *Candida albicans, Cryptococcus* spp. (Rella et al. 2012; Morales et al. 2013), and bacterial pathogens, e.g. methicillin-resistant *Staphylococcus aureus, Chlamydia* spp. (Li et al. 2018; Leanse et al. 2021). Kasozi et al. (2011) showed the antimalarial properties of PYO that could expand the potential uses of this compound to protozoan parasites. However, the authors concluded that the tested concentration of PYO in murine models (100 mg/kg) was toxic and cannot be applied in therapy, rather than the dose of 750 µg/mL presented by Hamad et al. (2020) that did not cause observable toxicity in tested rodents. PYO potential was also demonstrated in cancer research, where the viability of liver, pancreas, breast, lung, cervix, and skin cancer cell lines was reduced by this potential drug (Zhao et al. 2014; Patil et al. 2017; Moayedi et al. 2018; Abdelaziz et al. 2022). PYO may be used to produce other substances expressing anticancer properties. Moreover, Kohatsu et al. (2020) showed that IC_50_ of PYO and its halogenated form – chloropyocyanin, is much lower in human lung cancer and leukaemia cell lines than in normal fibroblasts. Patil et al. (2022) applied *P. aeruginosa* metabolites, namely PYO, and pyoverdine, to synthesize gold and silver nanoparticles that had cytostatic properties against Hep-G2, SK-MEL-2, HeLa, and A-549 cell lines. On the other hand, Peruzzo et al. (2021) demonstrated that PYO can be used for mitochondrial disease treatment by restoring the correct function of respiratory complex III. The authors confirmed the beneficial action of PYO on fibroblasts and proved that effective PYO concentrations were not toxic to *Drosophila*, *Danio rerio*, and mice. Additionally, it was also shown that PYO and other phenazine derivatives (i.e. PCA) act as 5-lipoxygenase inhibitors by binding to the active site of the enzyme (Santha and Vishwanathan 2021). Such findings may be crucial in the treatment of inflammatory diseases. Moreover, it was recently described that PYO can lead to the induction of bacteriophages from the lysogenic to lytic cycle, which is essential in phage therapy (Jancheva and Böttcher 2021).

**Fig. 2:**
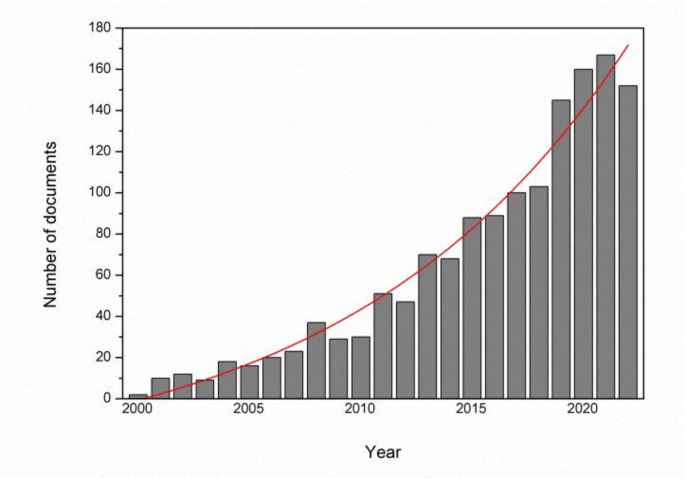
Scopus database analysis of documents including ‘pyocyanin’ as the searched phrase (years 2000–2022; search covering ‘pyocyanin’ found in the title, abstract, or keywords)

All the above-mentioned uses of PYO prove that apart from its contribution as a virulence factor, this compound may find numerous applications that will require intensified production. The growing demand can be confirmed by the number of studies using this pigment that are published. Over the last 30 years, PYO has gained more attention in the scientific community, which is confirmed by database analysis showing the rapidly growing number of articles (Fig. 2.). To date, several review articles focusing on PYO have been published (Pierson and Pierson 2010; Jayaseelan et al. 2014; Gonçalves and Vasconcelos 2021) and each of them underlines the potential applications of the pigment. Nevertheless, the analysis of PYO market prices lead to the conclusion that it remains a relatively expensive chemical since the prices vary from around €56.40 (calculated from $60) to more than €202.10 per 5 mg of PYO (Table 1.). PYO can also be chemically synthesized (Cheluvappa 2014; Kohatsu et al. 2019; Mortzfeld et al. 2019). However, up to now, the companies providing PYO as a chemical list *P. aeruginosa* culture as a source of the pigment (e.g. Sigma Aldrich). This indicates that biological production is a method of choice to introduce it to the market. Interestingly, based on the Scopus database search, the number of works showing PYO production in bioreactors is still scarce and usually focuses on electrical energy generation in MFCs.

**Table 1:** The analysis of the PYO market (price for 5 mg of the powder product, all prices were found on the company’s official website and calculated using the exchange rate of 15.12.2022 – €1.00=$1.07)

No.	Company	Price
1	A2B Chem	$154 (€143.93)
2	AA BLOCKS	$159 (€148.60)
3	Aaron Chemicals	$161 (€146.51)
4	Abcam	€100 ($107)
5	AK Scientific	$176 (€165.44)
6	APExBIO	$95 (€89.3)
7	AvaChem	$60 (€56.4)
8	Biomol	€98 ($104.86)
9	Focus Biomolecules	$60 (€56.4)
10	GLPBIO	$84 (€78.96)
11	Santa Cruz Biotechnology	€102 ($109.14)
12	Sigma Aldrich	€132 ($141.24)
13	Toronto Research Chemicals	$215 (€202.1)

## How to modulate pyocyanin production?

To date, multiple methods were proposed to modulate PYO production by *P. aeruginosa.* Most of them focus on the inhibition of pigment production due to its role as a virulence factor. Many substances inhibiting PYO production have been described. This group includes plant extracts (Naga et al. 2022; Inés Molina et al. 2022; Shariff et al. 2022), nanomaterials such as La_2_O_3_ nanoparticles (NPs), ZnO NPs, Sm_2_O_3_ NPs, Ag NPs and polysaccharide-capped Ag NPs, Ag-TiO_2_ and ZnO-graphene nanocomposites (Balusamy et al. 2012; Lee et al. 2014; Zanni et al. 2017; Alavi and Karimi 2018; Alavi et al. 2019; Saleh et al. 2019; Ali et al. 2020; El-Deeb et al. 2020; Zahmatkesh et al. 2022), antimicrobial peptides (calgranulin C) (Mishra et al. 2022), acylases (enzymes disrupting QS in bacteria) (de Celis et al. 2021), phenolic compounds (from olive oil processing waste) (Viola et al. 2022), nitric oxide (Gao et al. 2016), sodium citrate (Khayat et al. 2022), phage protein (gp70.1 from *P. aeruginosa* phage PaP3) (Zhao et al. 2016), hispidulin (flavone) (Anju et al. 2022), diallyl trisulfide (Li et al. 2022a), benzimidazolium salts (Önem et al. 2022), or co-cultivation with another microorganism (Liang et al. 2022). Zhou et al. (2022) proposed *phzM* gene as a target for reducing PYO production. Such approaches were proven to be effective against *P. aeruginosa* and inhibited PYO secretion. Among suggested mechanisms is inhibition of the expression of the genes involved in QS, which is intertwined, e.g., with PYO production and biofilm formation. Phenazine biosynthesis pathway starts from chorismate that is also a substrate for the synthesis of Pseudomonas quinolone signal (PQS), a QS signalling molecule. Furthermore, PQS may also mediate iron acquisition, another factor playing a role in PYO production (Lin et al. 2018). This creates the possibility for cross-reactions between these factors, leading to the inhibition of PYO synthesis.

On the other hand, more and more research is devoted to the intensification of PYO production, which is caused by the lack of a validated large-scale method in the industry. The currently used approaches focus on optimization of culturing conditions (e.g. agitation, pH, incubation time) and medium components, exposure to various chemical and physical factors or using genetic engineering methods to obtain a strain that is an effective PYO producer. Factors and approaches reported stimulating PYO production are summarized in Table 2.

**Table 2:** Reported factors stimulating PYO production

Adjustments made to the culture		Reference
Type	Adjustment	
Culture conditions and medium components	Addition of amino acids to the medium (tyrosine and valine)	(Sismaet et al. 2014)
	Optimization of culture conditions and medium ingredients	(Elbargisy 2021)
	Selection of carbon source	(Schmitz and Rosenbaum 2020)
	Co-culture with *Klebsiella variicola*	(Islam et al. 2018)
	Optimization of medium ingredients	(Preetha et al. 2007; Patil et al. 2017)
	Selection of carbon source and strain	(Bosire et al. 2016)
	Low phosphate content	(Whooley and McLoughlin 1982; Hassett et al. 1991; Matilla et al. 2022)
	Intermittent aeration of the culture	(Bagchi and Behera 2021)
Chemical compounds added to the medium (including nanomaterials)	Lanthanum oxide	(Balusamy et al. 2012)
	Multi-walled carbon nanotubes and zinc oxide nanoparticles	(Jabłońska et al. 2022a)
	Cerium oxide nanoparticles	(Xu et al. 2018)
	Silver nanoparticles	(Saeki et al. 2022)
	Gallium nitrate	(García-Contreras et al. 2014; Tovar-García et al. 2020)
	N-hexane	(Ozdal et al. 2019)
	Toluene	(Ozdal 2019)
	Sophorolipids	(Shen et al. 2014; Ajunwa et al. 2021)
	Ammonium chloride	(Allam et al. 2021)
	Manuka honey	(Mokhtar et al. 2020)
	Antibiotics in subinhibitory concentrations	(Shen et al. 2008; Cummins et al. 2009)
	Peptidoglycan and N-acetylglucosamine	(Korgaonkar and Whiteley 2011)
	Calcium chloride	(Sarkisova et al. 2005)
Physical factors	Static magnetic field	(Raouia et al. 2020)
	Static and rotating electromagnetic field	(Jabłońska et al. 2022b)
	Photoswitchable autoinducers and light λ = 365 nm (UV-A)	(Van Der Berg et al. 2015)
	Methylene blue and visible light	(Hendiani et al. 2019)
	Birnessite photoanode and visible light	(Ren et al. 2018)
	Hematite photoanode and visible light	(Ren et al. 2017)
Genetic engineering	Genetically modified *E. coli*	(da Silva et al. 2021)
	Genetically modified *P. putida*	(Schmitz et al. 2015; Askitosari et al. 2019)
	Overexpression of PqsE effector in *pqsC* deficient mutant	(Wang et al. 2013)
	Overexpression of *phzM*	(Yong et al. 2014)
	*rpoS*-deficient mutant of *P. aeruginosa*	(He et al. 2019)
	Overexpression of *rhl*	(Yong et al. 2011)
	Combined overexpression of *nadD* and *nadC* genes	(Ajunwa et al. 2022)
	*rpoS*-deficient mutant with overexpression of *phzM*	(Wang et al. 2020)

It is worth underlining that the choice of a good bacterial PYO producer is one of the vital factors in the upstream part of the bioprocess (Askitosari et al. 2019). A significant number of articles published used isolates from samples of water, soil, wastewater, or patients (El-Fouly et al. 2015; Patil et al. 2017; DeBritto et al. 2020; Ajunwa et al. 2022). However, this approach can make it difficult to use the strain in possible further research as they are often not deposited in any international and easily accessible collection of microorganisms such as American ATCC, British NTCC, German DSMZ, or Polish PCM. Surprisingly frequently, the strain of choice is *P. aeruginosa* PAO1. Nevertheless, some works reported that it is a poor PYO producer when compared with other strains (Dietrich et al. 2006; Bosire et al. 2016; Cao et al. 2017). Some authors proposed the use of PA14 strain (Price-Whelan et al. 2007; Sismaet et al. 2014; Elbargisy 2021), and *P. aeruginosa* ATCC 27853 (Hendiani et al. 2019; Jabłońska et al. 2022a, b).

Another important aspect is the choice of the medium and culture conditions. PYO production is most frequently conducted in Luria-Bertani broth (LB), glycerol-supplemented Nutrient Broth (GSNB), Mineral Medium, Tryptic Soy Broth (TSB), and King A medium (Price-Whelan et al. 2007; Sismaet et al. 2014; El-Fouly et al. 2015; Cao et al. 2017). Many researchers proved that low phosphate content is crucial for PYO production (Whooley and McLoughlin 1982; Hassett et al. 1991; Matilla et al. 2022). It is worth mentioning that some research groups attempted utilising raw materials and waste for PYO production, e.g. brewing process and maize cooking waste, corn steep liquor, potato washing water, coffee, tea, molasses, cheese, grape seeds, taro leaves, pea pods, moss, cotton seed meal, olive wastes, vegetable frying oil, corn, soya bean, sweet potato, watermelon seeds and groundnut (El-Fouly et al. 2015; Teixeira et al. 2019; Bacame-Valenzuela et al. 2020; DeBritto et al. 2020, Kahraman and Karaderi 2021). However, pigment production in this approach is usually relatively low. Only El-Fouly et al. (2015) and Teixeira et al. (2019) reported higher PYO production in waste-supplemented than in the conventional medium (cotton seed and beer waste, respectively). Most authors use agitation for enhanced production since PYO synthesis requires oxygen presence. However, some reported better production in stationary cultures (El-Fouly et al. 2015; Jabłońska et al. 2022a). An optimal pH of the process was estimated to pH = 7–8 by many research groups (Elbargisy 2021; Abdelaziz et al. 2022). Furthermore, the optimal temperature of incubation differs among the strains, and the most used values are within the range of 28–37℃.

Finally, it is more and more popular to utilise mathematical methods, e.g. statistical planning of experiment (Design of Experiment), to determine which factor significantly influences the obtained results and to optimize the process (Preetha et al. 2007; Patil et al. 2017; Bacame-Valenzuela et al. 2020).

The improvement of PYO production can also be achieved by the addition of chemical agents to the medium. Among them are nanomaterials, including the ones that were proven to inhibit PYO production. Nevertheless, the stimulative effect of ZnO NPs, Ag NPs, CeO_2_ NPs, and multi-walled carbon nanotubes was reported (García-Lara et al. 2015; Xu et al. 2018; Saeki et al. 2022; Jabłońska et al. 2022a). García-Lara et al. (2015) showed that the effect of ZnO NPs may be strain-dependent, as four out of 18 tested strains expressed an increased pigment production, whereas for the rest the result was the opposite. Saeki et al. (2022) reported that low concentrations of Ag NPs led to the upregulation of some QS regulatory genes. These findings were supported by Xu et al. (2018). Sophorolipids, organic solvents, some salts, antibiotics, and other substances were also reported enhancing PYO production (Table 2.). Another approach tested the influence of physical factors, e.g. electromagnetic field or light exposure (at different wavelengths, sometimes combined with chemical substances) on pigment secretion. The research suggests that the use of such agents can elevate the production in some cases and the suggested mechanism is linked to oxidative stress generated by the stressor (Hendiani et al. 2019). However, the mechanisms underlining these observations remain unclear.

A distinctively followed method of elevating PYO production during the past ten years seems to be genetic engineering of the strains. Thanks to the extensive knowledge of the biochemical pathways and molecular mechanisms engaged in PYO production, it is possible to target specific genes, either to knock them out or to enhance their expression. Chen et al. (2020) proved that *pip* gene positively regulates the expression of the *phz2* operon and therefore may be a targeted sequence to enhance PYO production. Xu et al. (2005) showed that the inactivation of *ptsP* gene leads to the overproduction of PYO in *P. aeruginosa* PA68 strain. The authors discovered that the lack of enzyme encoded by *ptsP* increased the activity of *lasI* and *rhlI* promoters, confirming that PYO and rhamnolipid production are connected, where more intensive production of one causes the inhibition of the other. Ajunwa et al. (2022) showed that overexpression of NAD synthase genes – nicotinic acid mononucleotide adenyltransferase (*nadD*) and quinolic acid phosphoribosyltransferase (*nadC*), led to increased PYO production in comparison to the wild-type strain. Wang et al. (2020) constructed a rpoS-deficient mutant and proved that the knockout led to higher *phzM* expression and an increase in PYO production.

Another approach presented in the literature is the insertion of the genetic constructs harbouring genes responsible for PYO production into other bacterial hosts, e.g., *Escherichia coli* or *Pseudomonas putida* (Schmitz et al. 2015; Askitosari et al. 2019; da Silva et al. 2021). The use of genetically engineered *E. coli* could be the best way of obtaining high PYO concentrations, as its generation time is much shorter than *P. aeruginosa* and the stationary phase is reached faster. However, the yield in the case of *E. coli* is still far from the best producers among Pseudomonads. Moreover, *E. coli* is less resistant to high PYO concentrations than *P. putida* (Askitosari et al. 2019), which possibly makes the latter the most promising strain for possible industrial use. The last possible future solution could be the generation of highly attenuated *P. aeruginosa* strain, as it was done by Valentine et al. (2020) in case of alginate production, or using a less virulent strain, such as *P. aeruginosa* ATCC 9027 that was confirmed by Grosso-Becerra et al. (2016) to be sensitive to antibiotics and avirulent in the murine model.

## Conclusion

The ambiguous nature of PYO makes it a good research candidate. It is well-known for its negative role, and further attempts to lower the virulence of *P. aeruginosa* should be continued. Notwithstanding, PYO should also be a recognized potent chemical with a wide variety of potential applications. Considering both these views, the research focused on its efficient and cost-effective production should be further conducted.

Based on the presented data, the market for PYO (and phenazines in general) is expected to grow over the following decades. The hallmarks of these developments are already visible with the launch of some commercial products based on PCA, that are already available on the market and can be applied in agriculture. Thus, an efficient and low-cost production technology should be sought and introduced. As we have shown, many approaches are tested to achieve this goal. To assure safety and maintain the process quality, it appears that genetically modified strains could be the most suitable candidates. However, the optimization methods for existing *Pseudomonas* models cannot be excluded, since they are still the most potent producers in the current state-of-the-art. There is considerable production potential that can be achieved through optimization techniques since many environmental factors can influence PYO production. Possibly, the PYO production can also be achieved by utilizing waste and raw products. Such attempts play along with green chemistry and circular economy ideals. Nevertheless, composition variabilities in these substrates can hinder obtaining a comparable product yield, which remains a challenge.
